# Comparison Patients and Staffs Satisfaction in General Versus Special Wards of Hospitals of Jahrom

**DOI:** 10.5539/gjhs.v7n6p95

**Published:** 2015-04-03

**Authors:** Leila Taheri, Marzieh Kargar Jahromi, Mohsen Hojat

**Affiliations:** 1 Faculty of Nursing and Paramedical, Jahrom University of Medical Sciences, Jahrom, Iran

**Keywords:** satisfaction, patient, staff, general ward, specific ward

## Abstract

**Introduction and Aims:**

Patient satisfaction is the most important indicator of high-quality health care and is used for the assessment and planning of health care. Also, Job satisfaction is an important factor on prediction and perception of organizational manner. The aim of this study is to identify and compare patient and staff satisfaction in general versus special wards.

**Material and Method:**

In order to identify the various indicators of satisfaction and dissatisfaction, a descriptive study (cross sectional) was done to assess patients’ satisfaction with in-patient care at Jahrom University of Medical Science hospitals. The sample size was 600 patients that selected by sequential random sampling technique and are close to their discharge from the hospital. Patients were asked to indicate the scale point which best reflected their level of satisfaction with the treatment or service. Also we assess the staff satisfaction (sample size was 408 staffs) in general ward using a researcher made questionnaire. It should be noted that the participants were anonymous and there was no obligation to participation. We tried to set a secure and comfortable environment for filling out the questionnaire.

**Results:**

Among 600 patients, 239 (n = 38.67%) were men and 368 (61.33%) were female. Number of nurses was 408, of which 135 (33.08%) were men and 273 (66.92%) female. There was a significant correlation between working experience and professional factors of personnel. The mean total patient satisfaction in general and special wards is (2.75 ± .35, 3.03 ± .53) respectively. Differences of patient satisfaction in domains such respect, care and confidence in general wards versus special ward were statistically significant, but there was no difference in expect time of patients in these wards. Differences Between the mean patient and staff satisfaction in the general wards versus special wards were statistically significant using independent t-tests (p = .018, p = .029). Spearman test showed a statistically significant correlation between patient and staff satisfaction (p = .044).

**Conclusion:**

For improving quality of medical services and effective functioning needs maximizing efforts to obtain full patient and staff satisfaction.

## 1. Introduction

The measurement of patient satisfaction is becoming increasingly popular because of its role in quality assurance and continuous quality improvement systems (Glajchen & Magen, 1995).

In improving the service delivery in hospitals, there is a need to place high priority on the patient and their level of satisfaction with the provided services (Murphy, Hastings, Izui & Peisert, 2013).

Greenslade suggests that managers and nurses play a role in promoting patient satisfaction. By focusing on creating a good climate for service, health care managers can enhance nursing performance and patient satisfaction (Greenslade & Jimmieson, 2011). Patient satisfaction has many benefits and should be considered a component of the care-delivery paradigm (Locke, Stefano, Koster, Taylor & Greenspan, 2011). Bjertnaes concluded that the most important predictors for overall patient satisfaction with hospitals are expectations and patient-reported experiences (Bjertnaes, Sjetne, & Iversen, 2012). Thus is necessary to pay attention to the quality of effective function and medical services, maximizing efforts to achieve full patient satisfaction (Szyca, Rosiek, Nowakowska & Leksowski, 2012). Patient satisfaction is the most important indicator of high-quality health care and is used for the assessment and planning of health care. There is a positive correlation between patient satisfaction and nursing care (Dzomeku, 2012).

Job satisfaction of nurses is closely related to working conditions and the organizational environment (Szyca, Rosiek, Nowakowska & Leksowski, 2012). Job satisfaction, is defined as the gladness or emotional satisfaction that the worker obtains as a result of the evaluation about his/her work (Gokçen, Zengin, Oktay, Alpak & Yildirim, 2013). Kovner et al., found that factors such as work and family conflicts, distributive justice, promotional opportunities, variety of work, supervisor support, autonomy, and organizational constraints are affected by nurse job satisfaction (Ajeigbe, Mcneese-Smith, Phillips, Leach, 2014). Studies from other countries indicate that using the results obtained from satisfaction surveys can have a profound effect on the quality of services (Soleimanpour et al., 2011).

Patient satisfaction evaluations can address:

1). The reliability of services, or the assurance that services are provided in a consistent and dependable manner;
2). The responsiveness of services or the willingness of providers to meet patient needs;
3). The courtesy of providers; and
4). The security of services, including the security of records.

Patient satisfaction surveys are most useful when they are designed to meet specific objectives and when they use appropriate methods and measures (Chiu, Cheng, & Hsieh, 2010). According to author researches, this is the first study that compare patient and staff satisfaction in general and special wards of hospitals in Iran.

## 2. Material and Methods

### 2.1 Setting

Hospitals of Jahrom University of Medical Science

### 2.2 Data Collection

The instruments used for the study were the first: Questionnaire for patient satisfaction contained 40 questions in five areas of respect, care, waiting times, outcomes and trust. Questionnaires administration was done by reading out and explaining the content to those who could not read and write. Those who could read and understand answered the questions independently. A simple five-point Satisfaction Scale was used, with 0 - 4 indicating the lowest and highest levels of satisfaction, respectively.

The second questionnaire was Staff satisfaction questionnaire containing 27 questions, 4 questions about demographic characteristics, and the rest were in four areas of job satisfaction: professional factors, payment system pioneer and welfare criteria.

The original questionnaire is validated by ten professors of nursing and paramedical college in Jahrom University of medical sciences. In the next step, to obtain the reliability of the questionnaire ten nursing staff was chosen to fill out the questionnaire twice with three weeks interval. Statistical analysis showed that the Cronbach α coefficient of the questionnaire was 0.86 and the performed test-retest had an appropriate reliability (r=0.81).

### 2.3 Intervention

In order to identify the various indicators of satisfaction and dissatisfaction, a descriptive study (cross sectional) was done to assess patients’ satisfaction with in-patient care at Jahrom University of Medical Science hospitals.

The sample size was 600 patients that selected by sequential random sampling technique and are close to their discharge from the hospital. Patients were asked to indicate the scale point which best reflected their level of satisfaction with the treatment or service.

Also we assess the staff satisfaction (sample size was 408 staffs) in general ward using a researcher made questionnaire. It should be noted that the participants were anonymous and there was no obligation to participation. We tried to set a secure and comfortable environment for filling out the questionnaire.

The study population inclusion criteria includes adult males and females of 18 years and above admitted into the general wards (medical, surgical and maternity) and special wards (ICU, CCUs, Hemodialysis, Cooley´s ward) of the hospitals for at least 24 hours. The consent of the patients was sought and those who met the inclusion criteria were given the questionnaires.

### 2.4 Data Analysis

For interpreting data on both the tools, it was calibrated using a 5-item Likert, below. The data after collecting were analyzed by SPSS version 16. The data were examined using percent, mean and standard deviation and chi-square and Spearman tests.

**Table:** 

Very high	High	intermediate	low	Very low
4	3	2	1	0

## 3. Results

Among 600 patients, 239 (n = 38.67%) were men and 368 (61.33%) were female.299 respondents had education degree lower than diploma (49.83%). 301 respondents (17.50%) hospitalized before.299 patients (83.49%) was their first time of hospitalization in this hospital.288 patients (38%) of the total respondents were encounter with a shortage of bed. Average length of stay in hospital was (2.35 ± 36.4) days (Table 1).

**Table 1:** Correlation between demographic variables and satisfaction factors of patients

Variable P value	respect	care	confidence	Expect time	outcome	Total satisfaction
**Gender**	0.41	0.88	0.48	0.58	0.82	**0.62**
**Previous hospitalization**	0.23	0.34	0.033	.074	.054	**.014**
**Age**	0. 33	0.22	0.19	0.12	0.62	**0.29**
**Length of stay in hospital**	0.67	0.76	.029	0.71	0.44	**0.52**

Number of personnel was 408, of which 135 (33.08%) were men and 273 (66.92%) female. 75% of people aged between 35-20 years and the average age was (33 ± 1.2) years. 300 personnel were official employee and rest of all were contractual and committal. Overall, 306 (75%) have been under 15 years of work experience and 102 (25%) over 15 years of working experience. Statistical analysis of the measured variables such as age, type of employment, gender and job satisfaction show that, correlation between job satisfaction and these various items using chi-square and Spearman tests is not statistically significant. There was just a significant correlation between working experience and professional factors of personnel (Table 2).

**Table 2:** correlation between demographic variables and job satisfaction factors of personnel

Variable P value	Professional	Payment	Pioneer	welfare	Total job satisfaction
**Gender**	0.25	0.86	0.18	0.34	**0.14**
**Type of employment**	0.99	0.82	0.96	0.90	**0.91**
**Age**	0.85	0.83	0.82	0.46	**0.94**
**Working experience(year)**	0.02	0.89	0.49	0.62	**0.13**

The mean total patient satisfaction in general and special wards is (2.75 ± .35, 3.03 ± .53) respectively. Differences of patient satisfaction in domains such respect, care and confidence in general wards versus special ward were statistically significant, but there was no difference in expect time of patients in these wards (table 3).

**Table 3:** Patient and staff satisfaction in general versus special wards

(P value)	Total	General	Special	Ward	
		
	SD± Mean			variables	
0.045	39.3±53.0	70.3±56.0	71.3±51.0	respect	Patients satisfaction
0.020	37.3±33.0	95.2±45.0	79.3±22.0	Care	
0.038	77.2±58.0	52.2±50.0	20.3±66.0	confidence	
0.928	74.2±30.0	74.2±12.0	74.2±49.0	Expect time	
0.031	20.2±23.0	51.2±13.0	89.1±34.0	Out come	
0.018	89.2±44.0	75.2±35.0	03.3±53.0	Total	
0.031	89.1±25.0	14.1±38.0	65.2±12.0	Professional	Personnel satisfaction
0.082	16.2±11.0	21.2±08.0	11.2±14.0	Payment	
0.037	36.3±52.0	89.2±51.0	83.3±54.0	Pioneer	
0.076	51.1±28.0	63.1±29.0	59.1±27.0	Welfare	
0.029	25.2±28.0	96.1±31.0	54.2±26.0	total	

Also staff satisfaction in general and special wards is respectively (1.96 ± .31, 2.54 ± .26), which were assessed as moderate. Job satisfaction in two domains profession and pioneer unlike payment and welfare was statistically different in general wards in comparison with special wards (Table 3).

Differences Between the mean patient and staff satisfaction in the general wards versus special wards were statistically significant using independent t-tests (p = .018, p = .029) (Table 3).

Spearman test showed a statistically significant correlation between patient and staff satisfaction (p = .044).

## 4. Discussion

Job satisfaction is an important factor in prediction and perception of organizational manner and any study about that would help managers to identify latent problems. Most studies on stress and job satisfaction in nursing have focused on general nursing specialties, and relatively little attention has been paid to compare satisfaction of nurses working in special versus general wards

In this article correlation between patient satisfaction and various items such as age, gender and previous hospitalization, is not statistically significant by using chi-square and Spearman tests. Alotaibi.et al concluded that in Kuwait overall satisfaction was higher among men than women, and it was also higher among those with university degree of education than the other levels of education (Rouhi, Asayesh, Rahmani & Abbasi, 2011), this result is inconsistent with the findings of the present study.

Based on our findings, correlation between age, type of employment and gender with job satisfaction domains, unlike working experience with professional factor domain, is not statistically significant. But Ahmadi et al studied 100 nurses that were randomly selected among those working in emergency, orthopedic, dialysis wards and intensive care unit (ICU). They found that the workplace in the hospital and gender of the nurses had an important role in their occupational burnout (Rouhi et al., 2011).

Patient satisfaction has been studied extensively using quantitative and qualitative methods. The results of all these studies revealed that nursing care was the major determinant of patient satisfaction. The emerging health care literature suggests that patient satisfaction is a prominent concern in the health services that is interlace with strategic decisions (Dzomeku, 2012).

The results of this study indicate that most of the patients interviewed were satisfied with the services they received in these two hospitals. This is in line with findings of patient satisfaction surveys elsewhere. A very low proportion of patients expressed dissatisfaction with various aspects of the services, especially with outcome and waiting times. Lioh, et al in Nigeria showed that the overall patients satisfaction with the quality of care was good, despite other domains of dissatisfaction such as cost of services, waiting time and hospital bureaucracy (Iloh et al., 2013).

You, et al through one study in 181 Chinese hospitals, concluded that 45% were dissatisfied with their jobs. Substantial percentages of nurses described their work environment and the quality of care on their unit as poor or fair (61% and 29%, respectively) (You et al., 2013). Zhou et al conducted a survey in 2013 on 350 Chinese medical staff. Their result shows that the overall satisfaction with the medical record service was high, 38.9% of participants considered it to be very satisfactory (Zhou, He, & Gao, 2013). According to Haghighizadeh findings, job satisfaction in the staff of Razi hospital in Iran was low and only one third of staff were satisfied by their jobs (Haghighizadeh, Daryaeepoor, Ghasemzadeh & Zahiri, 2013). In America, Eliadi suggests that nurses were dissatisfied with the relationships with the unit doctors, which indicates that the quality of professional relationships influences nurses’ satisfaction (C.A E, 1990). It seems that according to the country or hospital situation or cultural differences, the rate of satisfaction is conversional.

Emadi et al. (2010) concluded that critical care nurses were significantly less satisfied than general ward nurses with many aspects of their job (Emadi, Falamarzi, Al-Kuwari, & Al-Ansari, 2009). this study is not similar to our findings. Rouhi et al in Iran (2011) designed a descriptive & analytical study on 750 nurses and founded that intensive care and emergency wards’ nurses had the lowest organizational commitment and job satisfaction (Rouhi, Asayesh, Rahmani & Abbasi, 2011) that is consistence with our study. As with all research, this study had several limitations. First, job satisfaction was measured by single scale. This may have resulted in the loss of important information not gathered by the scale. Additionally, in order to facilitate a thorough understanding of factors underlying the relationship between personnel and patient satisfaction identified in this study, the cultural context and factors within a specific health care system must be considered.

## 5. Conclusion

Health care manager could be recommended as a comprehensive and suitable strategy to provide health services in public health. Paying attention to patient satisfaction is a basic step for quality improvement and should be done intermittently. For improving quality of medical services and effective functioning we need maximizing efforts to obtain full patient and staff satisfaction.

## References

[ref1] Ajeigbe D, Mcneese-Smith D, Phillips L, Leach L (2014). Effect of nurse-physician teamwork in the emergency department nurse and physician perception of job satisfaction. J Nurs Care.

[ref2] Bjertnaes Oa, Sjetne Is, Iversen Hh (2012). Overall patient satisfaction with hospitals: Effects of patient-reported experiences and fulfilment of expectations. Bmj Quality & Safety.

[ref3] (1990). C.A E. Nurse-physician collaboration and its relationship to nurse job stress and job satisfaction: University of Massachuesetts.

[ref4] Dzomeku V. E (2012). In patient satisfaction with nursing care: A case study at kwame nkrumah university of science and technology hospital. International Journal of Research in Medical and Health Sciences.

[ref5] Emadi N.A, Falamarzi S, Al-Kuwari M. G, Al-Ansari A (2009). Patients’ satisfaction with primary health care services in Qatar. MEJFM.

[ref6] Gokcen C, Zengin S, Oktay M, Alpak G, Yildirim C (2013). Burnout, job satisfaction and depression in the healthcare personnel who work in the emergency department. Anadolu Psikiyatri Derg.

[ref7] Greenslade J, Jimmieson N (2011). Organizational factors impacting on patient satisfaction: A cross sectional examination of service climate and linkages to nurses’ effort and performance. International Journal of Nursing Studies.

[ref8] Haghighizadeh M, Daryaeepoor S, Ghasemzadeh R, Zahiri M (2013). The relationship between Job satisfaction and burnout among rehabilitation personnel of Razi psychiatric hospital in Tehran. 2013.

[ref9] Iloh G.U, Ofoedu J, Njoku P, Okafor G, Amadi A, Godswill-Uko E (2013). Satisfaction with quality of care received by patients without national health insurance attending a primary care clinic in a resource. Poor environment of a tertiary hospital in eastern Nigeria in the Era of scaling up the Nigerian formal sector health In. Annals of Medical And Health Sciences Research.

[ref10] Locke R, Stefano M, Koster A, Taylor B, Greenspan J (2011). Optimizing patient caregiver satisfaction through quality of communication in the pediatric emergency department. Pediatric Emergency Care.

[ref11] Muhondwa Mtl M, Mwangu N, Mbembati M. J (2008). Patient Satisfaction at the Muhimbili National Hospital in dar es salaam, Tanzania. East African Journal of Public Health.

[ref12] Murphy D, Hastings Da, Izui C, Peisert K (2013). Prescriptions For Excellence In. Jama.

[ref13] Rouhi G, Asayesh H, Rahmani H, Abbasi A (2011). Job satisfaction and organizational commitment among nursing staff: A study from golestan.

[ref14] Soleimanpour H, Gholipouri C, Salarilak S, Raoufi P, Vahidi R. G, Rouhi A. J (2011). Emergency department patient satisfaction survey in Imam Reza Hospital, Tabriz, Iran. Int J Emerg Med.

[ref15] Szyca R, Rosiek A, Nowakowska U, Leksowski K (2012). Analysis of factors influencing patient satisfaction with hospital treatment at the surgical department. Polish Journal of Surgery.

[ref16] You L-m, Aiken L.H, Sloane D.M, Liu K, He G-p, Hu Y (2013). Hospital nursing, care quality, and patient satisfaction: Cross-sectional surveys of nurses and patients in hospitals in China and Europe. International Journal of Nursing Studies.

[ref17] Zhou Y, He Y, Gao H (2013). Survey of clinical medical personnel satisfaction and analysis of medical record service. Chinese Medical Record English Edition.

